# Hemophagocytic lymphohistiocytosis accompanying Still's disease: A case report

**DOI:** 10.1002/ccr3.7955

**Published:** 2023-10-10

**Authors:** Arman Ahmadzadeh, Neda Babadi, Faraneh Farsad, Saba Babadi, Shirin Assar

**Affiliations:** ^1^ Rheumatology Ward of Loghman Hakim Hospital Shahid Beheshti University of Medical Sciences Tehran Iran; ^2^ Department of Adult Rheumatology, Loghman Hakim Hospital, School of Medicine Shahid Beheshti University of Medical Sciences Tehran Iran; ^3^ Research Centre of Loghman Hakim Hospital Shahid Beheshti University of Medical Sciences Tehran Iran; ^4^ Department of Adult Internal Medicine Naft Grand Hospital Ahvaz Iran; ^5^ Clinical Research Development Center, Imam Reza Hospital Kermanshah University of Medical Sciences Kermanshah Iran

**Keywords:** adult‐onset Still's disease, hemophagocytic lymphohistiocytosis

## Abstract

Hemophagocytic lymphohistiocytosis (HLH) is a rare hematologic disease that occurs due to immune system dysfunction. Clinical manifestations of this disease are fever, increased ferritin level, cytopenia, and hemophagocytosis in the biopsy report of the bone marrow. We report a 36‐year‐old woman referred to our hospital with persistent fever, arthralgia in interphalangeal joints, and cutaneous rash on the trunk, was subsequently diagnosed as an adult‐onset Still's disease (AOSD), and after bone marrow aspiration, HLH was diagnosed with her.

## Graphical abstract

The salmon‐colored rash on the patient’s trunk who was diagnosed with Hemophagocytic Lymphohistiocytosis.

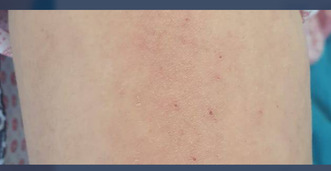

## 1 INTRODUCTION

Hemophagocytic lymphohistiocytosis (HLH) is a hyperinflammatory syndrome that is associated with a high mortality rate. HLH is characterized by hemophagocytosis and histiocytic proliferation.^1^ Primary HLH has a familial pattern and secondary HLH is reactive and also called macrophage activation syndrome (MAS). This type of HLH is usually acquired following autoimmune diseases, infection, and malignancy.^2^

The prevalence of HLH is not completely measurable because the diagnosis of this disease is difficult and there are other comorbid diseases at the time of diagnosis. In a study that was done in Sweden from 1987 to 2006, the prevalence of HLH was 1.5 per million.^3^

The most common manifestations of HLH are fever and cytopenia. The diagnosis of HLH starts with clinical suspicion of the physicians. The criteria for diagnosis of HLH include fever; splenomegaly; cytopenia (affecting at least two of three lineages in the peripheral blood); fasting triglyceride levels ≥3 mmol/L and/or fibrinogen level ≤1.5 g/L; serum ferritin level ≥ 500 ng/mL; CD25 level ≥ 2400 U/mL; decreased or absent natural killer (NK) cell activity; or hemophagocytosis in bone marrow, spleen, or lymph nodes. The diagnosis of HLH can be established if the patient fulfills at least five of the eight mentioned criteria.^4^

Adult‐onset Still's disease (AOSD) is an autoimmune disorder that can predispose patients to HLH. The coincidence of AOSD and HLH is rare.^5^

In this report, we present a woman with a coincidence of AOSD and HLH.

## 2 CASE PRESENTATION

A 36‐year‐old woman was referred to our hospital with chief complaints of fever and night sweating for about 2 months. The patient mentioned that she had pain in the interphalangeal joints of the both hands from about 1.5 years ago, after receiving COVID‐19 vaccine. She was referred to different physicians and oral corticosteroids and nonsteroidal anti‐inflammatory drugs (NSAIDs) were prescribed for her. She did not have fever, night sweating, pharyngitis symptoms, or skin rash at that time. She denied photosensitivity, malar rash, or Raynaud phenomenon. The patient had unintentional weight loss about 10 kg in less than 6 months.

**FIGURE 1:**
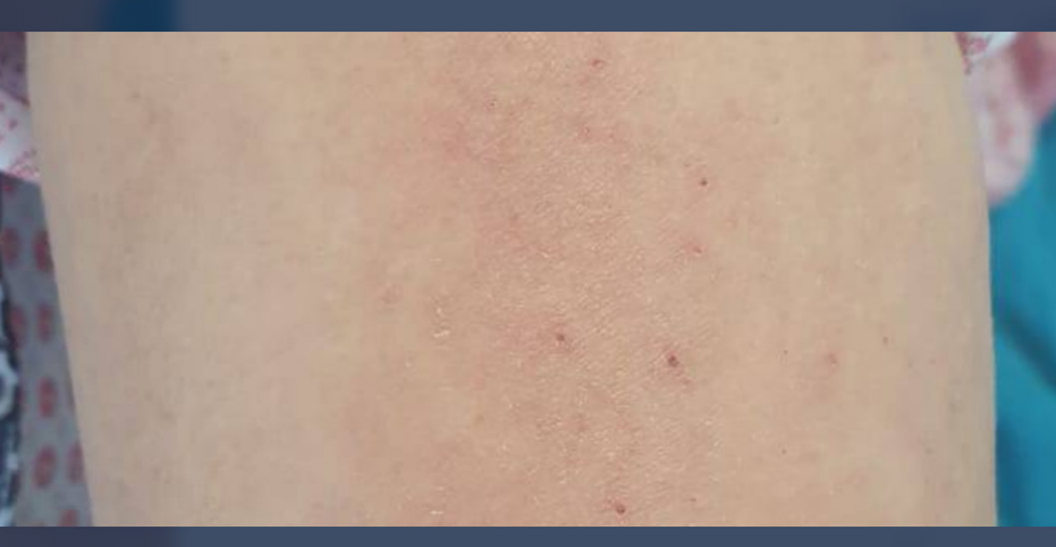
Showing the salmon‐colored rash on the patient's trunk.

On the first day of admission, the patient had reported fever, skin rashes, pain in knees, and little small joints of the hands since 2 weeks before referring to us and the fever responded to NSAIDs first, but after a few days, the fever did not respond to NSAIDs. There was no pain in back, pelvic, or chest. And also she did not have dyspnea. On the physical examination low‐grade fever (T = 37.7°^C^), red macular rashes (salmon‐colored rash), in the trunk without face involvement (Figure 1), and pharyngitis were detected. There was no obvious joint swelling. The examination of cardiovascular and respiratory systems was within normal limits.

She was previously evaluated for infectious diseases in another hospital. The infectious work‐ups included blood and urine cultures, serologic tests for hepatitis B and C viruses, and human immunodeficiency virus, and anti‐SARS‐CoV‐2 IgG and IgM antibodies, and COVID‐19 PCR tests which were all within normal limits. She had anemia (hemoglobin: 8 mg/dL) in evaluations and packed cell was prescribed for her. In that center, 2ME (2‐mercaptoethanol Brucella agglutination test), wright, coombs wright, antinuclear antibodies (ANA), anti‐double‐stranded DNA (anti‐ds‐DNA), anti‐citrulinated peptide antibody (anti‐CCP), human leukocyte antigen (HLA) B‐27 were checked and all were negative. All laboratory data are seen in Table 1. The patient was referred to our hospital for more evaluations due to persistent fever and rash.

**TABLE 1:** Laboratory findings of the patient.

Lad test (normal range)	First day	During hospitalization	After discharge
LDH (207–414 IU/mL)	1102	668	36
Ferritin (24–307 ng/mL)	9954	3300	740
CRP (up to 10 mg/dL)	80	156	6.5
AST (0–31 IU/L)	101	80	37
ALT (0–34 IU/L)	76	67	34
ALP (64–306 U/L)	676	530	334
WBC × 10^3^/mm^3^	17	16.2	6
Hemoglobin (12–14 mg/dL)	8.3	9.4	10.5
MCV (80–100 fL)	73	75	80
Platelet × 10^3^/mm^3^	119	251	369

In our center, due to persistent fever, pharyngitis symptoms, cutaneous rashes, digital arthralgia, and negative serology tests, Still's disease was diagnosed for the patient. Also, rheumatologists were suspicious to other differential diagnoses such as hemophagocytic lymphohistiocytosis (HLH) and hematological malignancies due to decreasing trends of blood cells. So, a hematologist evaluated her and she underwent bone marrow aspiration (BMA). The report of BMA confirmed HLH disease because hemophagocytosis was observed in her BMA specimen.

With this diagnosis, 4 mg of intravenous dexamethasone every 8 h was prescribed for her, improvement of clinical manifestations was started after 5 days and all blood parameters and blood ferritin had improving trends. Cyclosporine 50 mg/daily, and hydroxychloroquine 200 mg/daily were added 1 week after initiation of intravenous dexamethasone.

We were suspicious to malignancies and we requested chest and abdominal CT scans with contrast. In chest CT scan we observed mediastinum, supraclavicular, and axillary lymphadenopathy. There were no other significant findings in the chest and abdominal CT scans.

The patient was discharged with a good condition after 2 weeks of treatment by intravenous dexamethasone. She was treated with oral drugs (tab dexamethasone 0.5 mg/daily, tab cyclosporine 50 mg/daily, and tab hydroxychloroquine 200 mg/daily).

## 3 DISCUSSION

We presented a young female patient who had HLH secondary to AOSD. Her clinical and laboratory findings included fever, arthralgia, salmon‐colored rash, lymphadenopathy on chest CT scan, negative ANA, negative rheumatoid factor. She had hemophagocytosis. So she had five out of the eight diagnostic criteria of HLH. Then, the patient was diagnosed with HLH followed by AOSD.^6^, ^7^

Still's disease is an autoimmune disorder. Still's disease with adult‐onset (AOSD) has two ranges of presentation including 15–25 and 36–46 years of age.^8^ The etiology of this disease is not clear. Some factors including infections genetic factors, (viral or bacterial), and immune disorders are suggested.^8^

AOSD is characterized by fever, non‐suppurative pharyngitis, transient rash (salmon‐colored, maculopapular, nonpruritic, and often observed during febrile episodes) involving the trunk and proximal extremities, arthralgia that commonly involving the wrists, ankles, knees, and elbows.^8^ Our case had fever, pharyngitis, rashes with trunk involvement, and arthralgia especially in phalanxes.

Laboratory findings in AOSD are similar to other autoimmune disorders. Common laboratory findings are elevated white blood cell (WBC) count, abnormal LFTs, and elevated levels of CRP, and ferritin. In this disease, RF and ANA are usually negative. For diagnosis of AOSD, physicians should exclude infections disorders, vasculitis, malignancies, and other connective tissue disorders.^8^ We were suspicious to AOSD but we also excluded malignancies and infections disorders by different evaluations such as serologic tests, BMA, chest and abdominal CT scans.

Of the most serious complications of AOSD is HLH. AOSD and HLH have similar clinical findings and laboratory results. These similar features make a diagnostic challenge for physicians.^9^ HLH unlike to AOSD may have thrombocytopenia or leukopenia, and very high levels of triglyceride in serum.^10^ We observed significant elevation in LFT, LDH, ferritin, CRP, WBC levels. Thrombocytopenia and anemia (low level of hemoglobin) were observed in the laboratory tests of our case. We found high levels of serum ferritin in our case. It seems that a key to diagnosis AOSD is very high serum ferritin. Such a finding occurs in AOSD with HLH.^11^

Laboratory and clinical findings accompanying with decreasing trends of lineage blood cells suspected us to diagnose AOSD + HLH in the patient. This coincidence was previously reported.^12^, ^13^ Kuruvilla et al reported a 23‐year‐old male patient who presented with fever, joint pain, and rash for 20 days. On evaluation, he fulfilled the diagnostic criteria for AOSD and HLH, and a diagnosis of HLH secondary to AOSD was made. A previous review^13^ evaluated clinical characteristics and prognosis for 10 cases of AOSD complicated by HLH. They concluded that patients with AOSD complicated by HLH have worse prognosis and higher relapse rates compared to AOSD patients without HLH complications. High‐dose glucocorticoids (>1 mg/kg prednisone), and cyclosporine after 1–2 weeks of taking glucocorticoids were the most common therapeutic methods.

HLH after COVID‐19 vaccination was reported previously.^14^, ^15^ Though our patient‐reported vaccine injection before the initiation of arthralgia, but the interval between vaccine injection and development of anemia, skin rashes, and fever was more than 1 year. Therefore, we cannot consider an association between COVID‐19 vaccination and HLH development in our patient.

The treatment of HLH are corticosteroids and cyclosporine. Nonsteroid anti‐inflammation drugs and corticosteroids were recommended for treatment of AOSD. We used a combination of these treatments for our patient and she responded to these treatments and was discharged with a good condition.

## 4 CONCLUSION

Diagnosis of HLH accompanying Still's disease is important because it is rare and it may be misdiagnosed and can prone patients to mortality. The presence of fever, pharyngitis, arthralgia as clinical manifestations with high levels of blood inflammatory parameters including CRP, ferritin, and also increasing trends of lineage blood cell counts should suspect physicians to diagnose HLH + AOSD.

## AUTHOR CONTRIBUTIONS

**Arman Ahmadzadeh:** Conceptualization; writing – review and editing. **Neda Babadi:** Writing – original draft. **Faraneh Farsad:** Writing – original draft. **Saba Babadi:** Data curation. **shirin assar:** Writing – review and editing.

## FUNDING INFORMATION

We received no funding.

## CONFLICT OF INTEREST STATEMENT

The authors declare that they have no competing interests.

## ETHICS STATEMENT

Approval was not needed by the local Clinical Research Ethics Committee for case reports.

## CONSENT

Written informed consent was obtained from the patient for publication of this case report and any accompanying images. A copy of the written consent is available for review by the Editor‐in‐Chief of this journal.

## Data Availability

Data are available if requested.
